# Successful use of eculizumab in an 86-year-old patient with paroxysmal nocturnal hemoglobinuria in Japan

**DOI:** 10.1186/2193-1801-3-10

**Published:** 2014-01-04

**Authors:** Yokiko Ooe, Tomoko Nagai

**Affiliations:** Jutoku-kai Uegahara Hospital, 1-85 10 ban-cho Uegahara, Nishinomiya city, 662-0884 Hyogo, Japan; Department of Hematology, Hyogo Prefectural Tsukaguchi Hospital, 6-17 6-chome Minami-tsukaguchi-cho, Amagasaki city, Hyogo, 661-0012 Japan

**Keywords:** Renal function, Oldest Japanese patient, Paroxysmal nocturnal hemoglobinuria, Eculizumab

## Abstract

Eculizumab was used to treat an 86-year-old male patient with paroxysmal nocturnal hemoglobinuria, the oldest reported case in Japan. As observed in younger patients, this drug rapidly suppressed hemolysis in the present patient, which allowed weaning from blood transfusion. Eculizumab treatment has been continued for 2 years and resulted in the alleviation of renal dysfunction. Despite the patient’s advanced age, the inhibition of complement activity caused by this drug did not result in infection, indicating that it is safe to use in elderly patients.

## Background

The complement regulatory proteins CD55 and CD59 bind to glycosylphosphatidylinositol (GPI) on red blood cell membranes and inhibit complement activity, thus preventing hemolysis. Paroxysmal nocturnal hemoglobinuria (PNH) is a disease that involves hemolysis caused by the lack of CD55- or CD59-mediated complement activity inhibition in the absence of GPI anchor on the surface of red blood cells. This occurs in individuals with a defective phosphatidylinositol glycan class A enzyme that prevents normal GPI anchor synthesis (Takahashi et al. 1993). PNH is a very rare disease that develops in approximately 16 per 1 million people, and its onset does not exhibit any age specificity (Hillmen et al. 1995; Nishimura et al. 2004). Hemoglobin release into the bloodstream as a result of hemolysis could lead to massive nitrogen oxide (NO) scavenging in blood vessels; this increases platelet reactivity and aggregation, possibly inducing pulmonary hypertension and/or thrombosis. Hemolysis can also lead to several disorders including renal dysfunction due to massive hemosiderin deposition in the proximal renal tubules. The immune systems of elderly patients are generally compromised compared with those of younger patients (Naylor et al. 2005; Czesnikiewicz-Guzik et al. 2008). However, whether elderly patients with PNH can be safely treated using medicines that inhibit complement activity remains unknown.

We recently encountered a rare case of PNH diagnosed at the age of 81 years, in which eculizumab treatment was initiated at the age of 84 years. The details of the clinical course of this patient are presented herein.

## Case description

A 84-year-old man was diagnosed with anemia at a local facility in March 2009 and referred to a specialist. Upon examination at the facility, the patient was conscious, and his palpebral conjunctiva exhibited signs of anemia but no yellowing. His skin showed signs of anemia, but no purpura was detected. No superficial lymph nodes were palpable. No swelling of the liver or spleen, or significant heart or lung disorder was observed. The hematological test results were as follows: red blood cell (RBC) count, 241 × 10^4^/μL; hemoglobin (Hb), 8.4 g/dL; hematocrit, 26%; reticulocytes, 3.1%; and white blood cell (WBC) count, 3,370/μL (48.3% neutrophils, 41.2% lymphocytes, and 3.8% eosinophils). The biochemical test results were as follows: lactate dehydrogenase (LDH), 1,073 U/L; aspartate aminotransferase (AST), 54 U/L; serum creatinine (sCr), 1.49 mg/dL; blood urea nitrogen, 15 mg/dL; vitamin B12, >1,500 pg/mL; haptoglobin, <10 mg/dL; and direct Coombs test result, negative (Table 1).

**Table 1:** **Laboratory findings on diagnosis (March 6, 2009)**

Hematology		Biochemistry	
RBC	241 × 10^4^/μL	T-Bil	1.0 mg/dL
Hb	8.4 g/dL	AST	54 U/L
Ht	26%	ALT	16 U/L
MCV	107.9 Fl	LDH	1,073 U/L
MCH	34.9 pg	ALP	221 U/L
MCHC	32.3 g/dL	BUN	15 mg/dL
PLT	12.6 × 10^4^/μL	CRE	1.49 mg/dL
Reticulocyte	3.1%	CRP	<0.1 mg/dL
WBC	3,370/μL	Fe	76 μg/dL
Stab	0.0%	UIBC	127 μg/dL
Seg	48.3%		
Eosino	3.8%		
Baso	0.6%		
Mono	3.8%		
Lympho	41.2%		
E-bl	0.5%		

Bone marrow aspiration in April 2009 demonstrated hypercellular marrow: nuclear cell count (NCC), 21.3 × 10^4^/mm^3^; megakaryocytes (MgK), 53/mm^3^ with bilineage dysplasia (i.e., peroxidase-negative myeloid cells, mononuclear megakaryocytes, and increased total erythroblasts [58.8%]). Chromosome analysis revealed a karyotype of 45X-Y(4)/46XY(16). Therefore, the patient was diagnosed with myelodysplastic syndrome (MDS), i.e., refractory cytopenia with multilineage dysplasia. His anemia gradually progressed, and he occasionally required blood transfusion.

**Figure 1:**
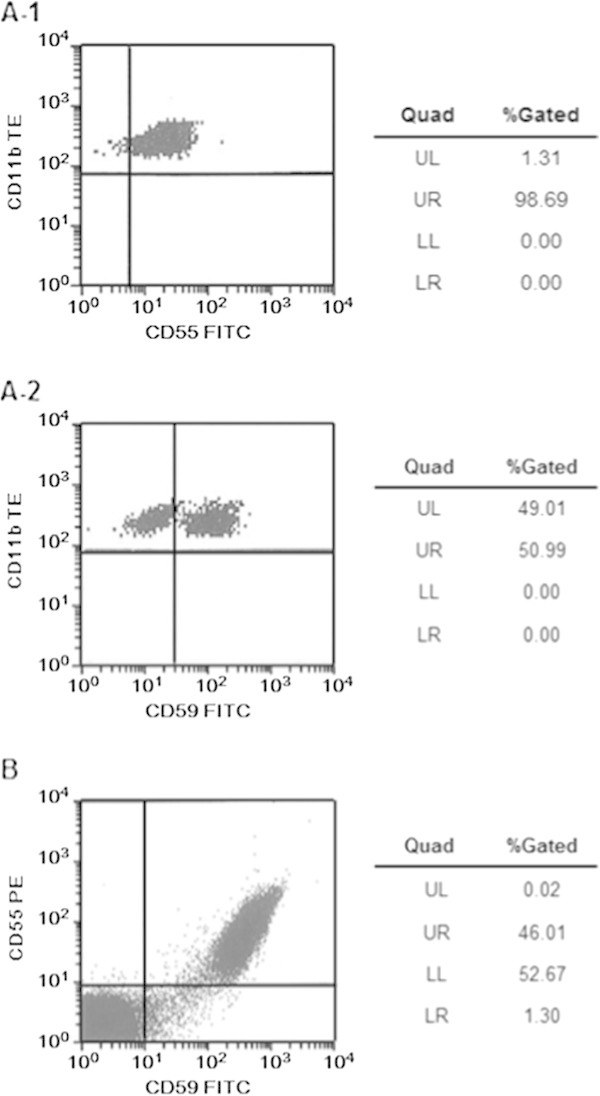
**Flow cytometry of red blood cells. A-1**: CD55 (pre-eculizumab). **A-2**: CD59 (pre-eculizumab). **B**: CD55/CD59 (post-eculizumab).

In November 2010, the patient’s urine output exhibited a cola color. Flow cytometric analysis of red blood cells at the time revealed 1.31% CD55(-) cells and 49.1% CD59(-) cells, prompting a diagnosis of PNH (Figure 1). In January 2011, infectious enteritis triggered hemolysis, which warranted blood transfusion. Oral treatment with methenolone 15 mg/day and prednisolone 10 mg/day was subsequently initiated, alleviating the patient’s dependence on blood transfusion. However, his hemolysis continued, and renal function gradually deteriorated; estimated glomerular filtration rate (eGFR) decreased from 35.9 mL·min^-1^·1.73 m^-2^ at the patient’s first visit to 25.2 mL·min^-1^·1.73 m^-2^. Therefore, eculizumab treatment was initiated in November 2011. Meningococcus vaccine was administered 2 weeks before eculizumab intervention.

Hematological tests performed immediately before eculizumab treatment initiation yielded the following results: RBC, 280 × 10^4^/μL; Hb, 10.0 g/dL; and reticulocytes, 4.1%. The biochemical test results were as follows: LDH, 1,748 U/L; and total bilirubin, 2.4 mg/dL.

**Figure 2:**
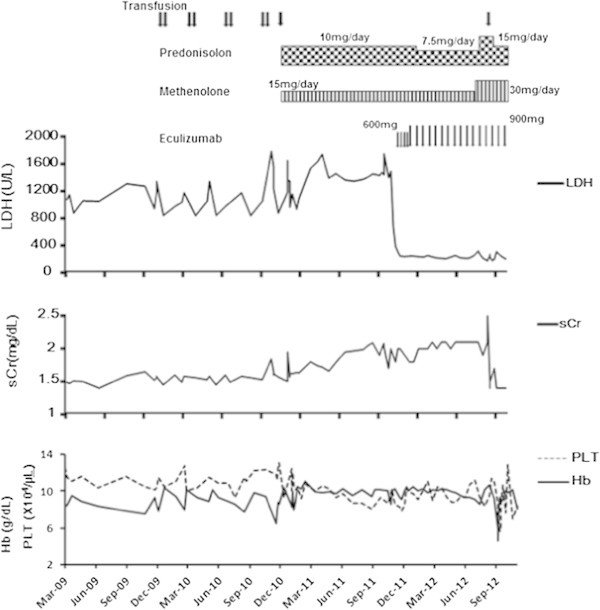
**Clinical changes observed in the patient.**

Eculizumab treatment rapidly reduced LDH levels, which returned to normal (i.e., 234 U/L) 4 weeks after treatment initiation and remained normal thereafter (Figure 2). In February 2012, the percentages of CD55(-) and CD59(-) cells increased to 53.97% and 47.31%, respectively (Figure 1). Ten months after eculizumab administration, the patient suddenly developed symptoms of anemia, which necessitated blood transfusion. However, this episode was not caused by PNH-induced hemolysis but rather by a bleeding ulcer from the remaining part of the stomach after previous gastric cancer surgery. The ulcer healed in response to fasting and treatment with a proton pump inhibitor and did not trigger a hemolytic attack. No infection-associated adverse events occurred.

Blood transfusion was no longer required thereafter. The patient experienced persistent suppression of hemolysis and renal function recovery, with sCr levels decreasing from 2.0 mg/dL (eGFR, 25.4 mL·min^-1^·1.73 m^-2^) to 1.4 mg/dL (eGFR, 37.5 mL·min^-1^·1.73 m^-2^).

## Discussion and evaluation

Patients with PNH associated with hemolysis are known to present with symptoms including abdominal pain, dysphagia, and pulmonary hypertension due to excessive NO scavenging by hemoglobin released into the blood or thrombosis at various sites as a result of stimulated platelet aggregation (Hill et al. 2010; Rother et al. 2005). Anemia secondary to hemolysis used to be treated with blood transfusion, steroids, or the anticoagulant warfarin (Parker et al. 2005; Hillmen et al. 2007). However, the use of eculizumab, which specifically inhibits complement C5, was approved in Japan in 2010. The complement regulatory proteins CD55 and CD59 play roles in hemolysis prevention. While CD55 regulates the first half of the complement activation pathway by stimulating the destruction of the C3/C5 converting enzyme, CD59 acts on C9 and inhibits the formation of the membrane attack complex (Nicholson-Weller et al. 1981; Sugita et al. 1988; Davies et al. 1989). When the present patient was diagnosed with PNH, the percentages of CD55(-) and CD59(-) cells were 1.31% and 49.1%, respectively. The sensitivity of the complement system increases particularly when CD59 is defective; this can lead to the hemolysis of PNH-affected blood cells. Therefore, this mechanism likely led to PNH development in the present case despite the lower percentage of CD55-negative cells detected. Eculizumab reduces LDH levels soon after treatment initiation (Hillmen et al. 2007). Furthermore, as hemolysis is alleviated by this drug, Hb levels gradually increase (Hillmen et al. 2007; Kanakura et al. 2011). The present patient was initially diagnosed with MDS and exhibited increased WBC counts in response to treatment with methenolone and prednisolone. However, because compromised renal function was still observed, the possibility of PNH-induced chronic renal failure and a subsequent poor prognosis was considered. Therefore, treatment with eculizumab was initiated. Compromised renal function is relatively frequent in patients with PNH. In the AEGIS Study, an indication study in Japan, as much as 66% of the patients studied were associated with abnormal renal function. Another study reports that 18% of Japanese patients with PNH died from renal failure (Nishimura et al. 2004). Furthermore, Japanese and global studies demonstrate improved renal function in 30–40% of all patients receiving prolonged eculizumab treatment (Kanakura et al. 2011; Hillmen et al. 2010). Therefore, eculizumab treatment could be effective for patients with PNH and compromised renal function.

In the present case, the LDH level immediately prior to treatment initiation was 1,491 U/L; it started decreasing soon after eculizumab treatment initiation, returning to normal (i.e., 234 U/L) 4 weeks later. Eculizumab protects red blood cells that lack CD55 or CD59 (also known as “PNH-type red blood cells”) from hemolysis, resulting in higher percentages of PNH-type red blood cells in patients with PNH (Hillmen et al. 2010). In fact, high percentages of PNH-type red blood cells were observed in the present patient after eculizumab treatment (54% and 47% of CD55(-) and CD59(-) cells, respectively). This suggests that PNH-type red blood cells survived and did not undergo hemolytic destruction.

In view of age-associated reduction in immune function and the effect of eculizumab on the suppression of the terminal complement pathway (i.e., C5 and subsequent components), one concern is that treatment with eculizumab may elevate the risk of infection in elderly patients (Naylor et al. 2005; Czesnikiewicz-Guzik et al. 2008). Indication studies of eculizumab have been performed globally including Japan. However, because of the nature of these studies, patients aged >80 years were rarely enrolled; the oldest patient enrolled in Japan was aged 70 years. The major adverse events observed in that study were headache (52%), nasopharyngitis (41%), nausea (21%), and diarrhea (13%); however, no infectious adverse events possibly associated with eculizumab treatment were reported (Hillmen et al. 2010). The present patient was diagnosed with PNH at the age of 81 years and began receiving eculizumab treatment at the age of 84 years. This is a rare case of an elderly patient treated with eculizumab. Moreover, the renal function of this patient improved from CKD stage 4 to stage 3 during eculizumab treatment.

## Conclusions

Our experience with the present case indicates that even patients aged >80 years with compromised renal function can be safely treated with eculizumab. The findings of this case suggest that eculizumab treatment is effective and useful for improving renal function, facilitating weaning from blood transfusion, and alleviating PNH patients’ fear of experiencing a hemolytic attack.

## Abbreviations

GPI: Glycosylphosphatidylinositol
PNH: Paroxysmal nocturnal hemoglobinuria
NO: Nitrogen oxide
RBC: Red blood cell
Hb: Hemoglobin
WBC: White blood cell
LDH: Lactate dehydrogenase
AST: Aspartate aminotransferase
sCr: Serum creatinine
NCC: Nuclear cell count
MgK: Megakaryocytes
MDS: Myelodysplastic syndrome
eGFR: Estimated glomerular filtration rate
CKD: Chronic kidney disease.
